# Supplementary material for: Content-rich biological network constructed by mining PubMed abstracts
Source: BMC Bioinformatics. 2004 Oct 8;5:147. doi: 10.1186/1471-2105-5-147 (PMC528731; doi:10.1186/1471-2105-5-147)
Supplement: Additional File 5 — The original Chilibot query results of the term "long-term potentiation (LTP)" and 22 other terms, limiting the latest references analyzed to the years 1990, 1995, 2000, and 2004. [file 1471-2105-5-147-S5.bz2 › chilibotAdditionalFile5/ltp2004/html/NMDA_TAU.html]

 


 **NMDA** and **TAU** 
  
Found 101 abstracts in PubMed,  **30 abstracts were retrieved and analyzed**.  


---

 Search Google  |
 PDF files only 
|  EDU domain only 

---

**Interactive relationship** (e.g. stimulation, inhibition, etc)

- The  **NMDA**  channel antagonist MK 801 reduced  **tau**  phosphorylation but not apoptosis after HC treatment, suggesting that HC mediated apoptosis was not due to calcium influx.  Ref: 12424737 J Neurosci Res, 2002
- Moreover, elevation of intracellular calcium via non  **NMDA**  receptor activation enhanced I  **Tau**  reversibly.  Ref: 12624748 Amino Acids, 2003
- Frequency ratio of  **NMDA**  versus AMPA sEPSCs in slices was correlated with the weighted time constant of decay  **tau**  w of  **NMDA**  sEPSCs and decreased with development along the reported decrease of  **tau**  w.  Ref: 11877500 J Neurophysiol, 2002
- The results showed that 1 extracellular Gln was low whereas Glu and  **Tau**  were high during infusion of  **NMDA**  0.5 1.0 mM or high K 80 mM in the hippocampus and ventrobasal thalamus, 2 hippocampal extracellular Glu, Gln and  **Tau**  were increased in response to the Glu uptake inhibitor, L trans pyrrolidine dicarboxilic acid tPDC, 0.5 3.0 mM, in a concentration dependent manner, 3 high K induced increase of extracellular Glu was partially blocked by the addition of 10 mM CoCl 2 with the high K dialysate in the hippocampus.  Ref: 10812202 Neurochem IntNeurochem Int, 2002

**Parallel relationship** (e.g. studied together, co-existance, homology, etc.)

- The roles of glycogen synthase kinase 3beta GSK 3beta and  **tau**  phosphorylation were examined in seven day old rats injected with the  **NMDA**  receptor antagonist MK801 that is known to induce neuronal apoptosis.  Ref: 11886446 Eur J Neurosci, 2002
- **NMDA**  receptor antagonists, MK 801 5 methyl 1 1 dihydro 5H dibenzo a, d cycloheptene 0 imine maleate, 10 microM and DL AP 5 DL 2 amino 5 phosphonovaleric acid, 250 microM, a nitric oxide synthase inhibitor, L nitroarginine L NNA, 500 microM, and an antioxidant, taurine  **Tau** , 10 mM, markedly attenuated the cell volume increasing effect of ammonia.  Ref: 12742072 Neurochem IntNeurochem Int, 2002
